# Supplementary material for: A novel oncolytic vaccinia virus with multiple gene modifications involved in viral replication and maturation increases safety for intravenous administration while maintaining proliferative potential in cancer cells
Source: PLoS One. 2025 Mar 6;20(3):e0312205. doi: 10.1371/journal.pone.0312205 (PMC11884718; doi:10.1371/journal.pone.0312205)
Supplement: Attachment — Submitted filename: Response to Reviewers.docx [file pone.0312205.s001.docx]

Dr. Milad Zandi

Academic Editor

PLOS ONE

December 22, 2023

**Manuscript reference number: PONE-D-23-30862**

**Title: Oncolytic vaccinia virus with multiple gene modifications increases safety while maintaining proliferative potential in cancer cells**

Dear Dr. Zandi:

Thank you for your email dated November 15, 2023, enclosing the reviewers’ comments. We appreciate your kind suggestion about resubmission and the constructive comments made by you and the reviewers. We have carefully reviewed the comments and have revised the manuscript accordingly. Our responses are given in a point-by-point manner below.

We hope the revised version is now suitable for publication and look forward to hearing from you in due course.

Go Okita

KM Biologics Co., Ltd.

**RESPONSES TO REVIEWERS’ COMMENTS**

**Journal requirements:**

*1. When submitting your revision, we need you to address these additional requirements.*

*Please ensure that your manuscript meets PLOS ONE's style requirements, including those for file naming.*

**Our responses:**

We prepared the submitted manuscript in accordance with 'MANUSCRIPT BODY FORMATTING GUIDELINES' and 'TITLE, AUTHOR, AFFILIATIONS FORMATTING GUIDELINES'. We ensured that our manuscript met the style requirements of PLOS ONE.

*2. Please confirm that the statement in the Competing Interests section does not alter your adherence to all PLOS ONE policies on sharing data and material. If there are restrictions on sharing of data and/or materials, please state these.*

**Our responses:**

Competing financial interests arise with respect to patents filed by the authors' institutions. Funding and competing interests are shown below.

Funding: This work was fully supported by KM Biologics Co., Ltd. All authors were employees of KM Biologics Co., Ltd. at the time of this study. The founder had no role in the study design, data collection and analysis, decision to publish, or preparation of the manuscript. The authors received no other specific funding for this work.

URLs to sponsors’ websites: https://www.kmbiologics.com

Competing interests: GO and TM are employees of KM Biologics Co., Ltd. KS and MS are employees of Meiji Animal Health Co., Ltd. In connection with this research, KS, GO, and MS are the inventors of a registered patent (JP7034818B2), TM and GO are the inventors of an applied patent (WO/2021/029385). These does not alter our adherence to PLOS ONE policies on sharing data and materials.

*3. Please include your full ethics statement in the ‘Methods’ section of your manuscript file. In your statement, please include the full name of the IRB or ethics committee who approved or waived your study, as well as whether or not you obtained informed written or verbal consent. If consent was waived for your study, please include this information in your statement as well.*

**Our responses:**

The submitted manuscript does not contain any studies involving human subjects.

Regarding the animal studies conducted, we have written an ethics statement in the Methods section.

*4. We note that Figure 2 and 9 in your submission contain copyrighted images.*

**Our responses:**

The photographs and descriptions used in the Figures 2 and 9 were taken and created by us ourselves in the laboratory of KM Biologics, Co., Ltd., and are used for the first time in this submitted manuscript. Therefore, these figures do not contain any copyrighted images.

**Comments from Reviewer #1**

*The strength of the paper is well structured, at all the work is of certain significance. The paper provides a very powerful message about the oncolytic vaccinia virus which used in this study as a gene insertion vector to arm immunostimulatory functions and is expected to be developed as a systemic oncolytic immunotherapeutic drug for patients with metastatic cancer. However, the study was needed to some modifications as following to be ready for publications. Please address the following comments in your consideration:*

*1-All figures in the manuscript will need to modifications; it is hesitated and not clear.*

**Our responses:**

Thank you for pointing out the quality of the figures. We re-created all figures and verified that the "Image Problems" were resolved using the Preflight Analysis and Conversion Engine (PACE) digital diagnostic tool.

*2-The manuscript will need to the statistical evaluation.*

**Our responses:**

Thank you for pointing out the need for statistical evaluation. This paper is our first research report on the creation of a novel oncolytic virus and focuses on vaccinia virus genome editing technology. Because we extensively evaluated trends in the properties of the viruses, we were unable to conduct experiments with a sufficient sample size for statistical analysis.

Since triplicate measurements were possible only in experiments for evaluating the cytotoxicity of each modified virus against cancer cells and normal cells, a t-test was performed on the difference in mean values. The results have been added to Results and Figure 3.

**Comments from Reviewer #2**

*Despite the authors' efforts, the study design is poor, and the study lacks studies such as cancer cell apoptosis and western blotting, which are necessary to confirm the results. For such studies, the proteins of the apoptotic pathway and their signaling should be examined. In addition, the topic of this manuscript is not a priority and the manuscript is boring, for example, there is no need to write such a long abstract.*

**Our responses:**

We would like to thank the reviewers for their comments on this point. This paper is our first research report on the creation of a novel oncolytic virus using vaccinia virus genome editing technology. It focuses on genome editing technology using chromosomes. Therefore, there may be insufficient knowledge regarding the mechanism of action of these viruses for their oncolytic effects and subsequent induction of antitumor immunity. In the future, we believe that it will be important to analyze the phenomena caused by this virus from cell biological and biochemical perspectives.

We followed the 'Submission Guidelines' and created a summary of the main objectives, methods, and most important results of this study in no more than 300 words. Thus, we would like you to understand that we will leave the original text as is.
