# Supplementary material for: A novel oncolytic vaccinia virus with multiple gene modifications involved in viral replication and maturation increases safety for intravenous administration while maintaining proliferative potential in cancer cells
Source: PLoS One. 2025 Mar 6;20(3):e0312205. doi: 10.1371/journal.pone.0312205 (PMC11884718; doi:10.1371/journal.pone.0312205)
Supplement: Attachment — Submitted filename: plos one.docx [file pone.0312205.s002.docx]

- The design of this study is good, but the big problem is why the authors considered SCID mice model and didn’t experiment on murine cancer model. It is more valuable if the authors investigate immune responses in immunocompetent mice model to evaluate modified vaccinia inoculation into in-vivo models for evaluating the potential of these modified vaccinia to induce antitumor immune responses.

- The abstract doesn’t include a conclusion relevant to the finding. Please reorganize the abstract according to the submission guideline.

- The title needs to mention more specifically, for example, indicate the unique modifications for the oncolytic vaccinia virus.

- In Fig. 9, the resolution of mice photographs is poor and viral symptoms induced by each modified virus are not indicated well.

- Please indicate labels for each bar chart for normal and Hela cells in Fig. 3.

- In Fig. 2, mock Hela cell is not shown to compare with other modified viruses infected Hela.

- There is a lack of statistical analysis section in the manuscript.

- In the method section, the authors didn’t mention what tests (MTT or ...) were used for evaluating oncolytic efficacy on various cells. Please explain about the cell viability assay you used in the method section and determine the cut-off for PFU/cell for determining notable cytotoxicity in normal and cancer cell line.
