# Supplementary material for: A novel oncolytic vaccinia virus with multiple gene modifications involved in viral replication and maturation increases safety for intravenous administration while maintaining proliferative potential in cancer cells
Source: PLoS One. 2025 Mar 6;20(3):e0312205. doi: 10.1371/journal.pone.0312205 (PMC11884718; doi:10.1371/journal.pone.0312205)
Supplement: Attachment — Submitted filename: Response to Reviewers_R2.docx [file pone.0312205.s003.docx]

Dr. Milad Zandi

Academic Editor

PLOS ONE

February 20, 2024

**Manuscript reference number: PONE-D-23-30862R1**

**Title: Oncolytic vaccinia virus with multiple gene modifications increases safety while maintaining proliferative potential in cancer cells**

Dear Dr. Zandi:

Thank you for your email dated January 15, 2024, enclosing the reviewers’ comments. We appreciate your kind suggestion about resubmission and the constructive comments made by you and the reviewers. We have carefully reviewed the comments and have revised the manuscript accordingly. Our responses are given in a point-by-point manner below. Other minor corrections are reflected in the ‘Revised Manuscript with Track Changes’ file.

We hope the revised version is now suitable for publication and look forward to hearing from you in due course.

Go Okita

KM Biologics Co., Ltd.

**RESPONSES TO REVIEWERS’ COMMENTS**

**Journal requirements:**

*1. Please review your reference list to ensure that it is complete and correct. If you have cited papers that have been retracted, please include the rationale for doing so in the manuscript text, or remove these references and replace them with relevant current references. Any changes to the reference list should be mentioned in the rebuttal letter that accompanies your revised manuscript. If you need to cite a retracted article, indicate the article’s retracted status in the References list and also include a citation and full reference for the retraction notice.*

**Our responses:**

　　The reference list has been revised as follows.

No. 7: “Stuart N Isaacs SN” to “Isaacs SN” (delete)

No. 12: “Anti-Tumor Effects of MAPK-Dependent Tumor-Selective Oncolytic Vaccinia Virus Armed with CD/UPRT against Pancreatic Ductal Adenocarcinoma in Mice.” to “Anti-tumor effects of MAPK-dependent tumor-selective oncolytic vaccinia virus armed with CD/UPRT against pancreatic ductal adenocarcinoma in mice.” (lowercase)

No. 20: “... in G cell cycle ...” to “... in G_1_ cell cycle ...” (add subscript)

No. 22: “Targeting Aberrant ...” to “Targeting aberrant ...” (lowercase)

No. 23: “Delivery and Biosafety of Oncolytic Virotherapy” to “Delivery and biosafety of oncolytic virotherapy.” (lowercase)

No. 24: “Mansun Law M” to “Law M” (delete)

No. 25: “Shen B.-Q” to “Shen B-Q” (delete)

**Additional Editor Comments:**

*- The design of this study is good, but the big problem is why the authors considered SCID mice model and didn’t experiment on murine cancer model. It is more valuable if the authors investigate immune responses in immunocompetent mice model to evaluate modified vaccinia inoculation into in-vivo models for evaluating the potential of these modified vaccinia to induce antitumor immune responses.*

**Our responses:**

In this study, we used SCID mice in which the influence of antiviral immunity was eliminated to evaluate the in vivo toxicity of the virus itself.

This paper is our first report and focuses on the construction of a novel recombinant oncolytic vaccinia virus and its safety confirmation in vitro and in vivo. As the editor commented, in the future, we believe that it is necessary to advance research on these viruses into cancer model animals and clinical settings, as well as cancer immunological analysis. We have added such a description to the ‘Discussion’ section. Furthermore, virus safety evaluation using SCID mice can be used as basic data for evaluation in model animals transplanted with human cancer cell lines.

*- The abstract doesn’t include a conclusion relevant to the finding. Please reorganize the abstract according to the submission guideline.*

**Our responses:**

We included the conclusion in the abstract and reorganized it according to the submission guideline.

*- The title needs to mention more specifically, for example, indicate the unique modifications for the oncolytic vaccinia virus.*

**Our responses:**

Following your instructions, we changed the title to:

‘A novel oncolytic vaccinia virus with multiple gene modifications involved in viral replication and maturation increases safety for intravenous administration while maintaining proliferative potential in cancer cells’.

*- In Fig. 9, the resolution of mice photographs is poor and viral symptoms induced by each modified virus are not indicated well.*

**Our responses:**

In order to improve the resolution of the photograph in Fig. 9, we decided to show only the results for the medium dose (5.0×10^5^ PFU) and PBS control.

All figures were converted and diagnosed using the PACE tool to meet PLOS requirements.

*- Please indicate labels for each bar chart for normal and Hela cells in Fig. 3.*

**Our responses:**

We indicated data labels for each bar chart in Fig. 3.

*- In Fig. 2, mock Hela cell is not shown to compare with other modified viruses infected Hela.*

**Our responses:**

The following description has been added to the ‘Results (Decrease in cytotoxicity in normal cells by the RNR gene deficiency)’ section:

‘No fluorescence was observed in mock HeLa cells that do not express EGFP (data not shown).’

*- There is a lack of statistical analysis section in the manuscript.*

**Our responses:**

We added 'Statistical analysis' subsection to the 'Materials and methods' section.

*- In the method section, the authors didn’t mention what tests (MTT or ...) were used for evaluating oncolytic efficacy on various cells. Please explain about the cell viability assay you used in the method section and determine the cut-off for PFU/cell for determining notable cytotoxicity in normal and cancer cell line.*

**Our responses:**

To evaluate the cytotoxic activity of the engineered viruses against cancer cells and their safety in normal cells, we infected HeLa and primary normal human skin fibroblasts with the viruses followed by Cell Count Kit-8 (CCK-8) was used to calculate the percentage of viable cells. CCK-8 contains a tetrazolium salt (WST-8) and an electron carrier (1-Methoxy PMS). CCK-8 solution was placed in each well of the cultured plates, and the absorbance at 450 nm was measured after 2 hours of incubation. Under these conditions, it was confirmed that there was a linear correlation between the number of viable cells and the absorbance. We have added these descriptions to the ‘Viral cytotoxicity’ subsection of the ‘Materials and methods’ section.

We had determined the infectious titer (PFU) of each virus solution using RK13 cells, and then adjusted the amount of virus to be inoculated in the cytotoxicity test. Since plaque formation tests can only be performed with special cells, cytotoxicity tests on various cells were measured using CCK-8, which can directly reflect the number of viable cells. As stated in the manuscript, the cell viability of each virus-inoculated group was quantified using CCK-8 with the absorbance of the control group without virus inoculation set to 100%.
