# Supplementary material for: A novel oncolytic vaccinia virus with multiple gene modifications involved in viral replication and maturation increases safety for intravenous administration while maintaining proliferative potential in cancer cells
Source: PLoS One. 2025 Mar 6;20(3):e0312205. doi: 10.1371/journal.pone.0312205 (PMC11884718; doi:10.1371/journal.pone.0312205)
Supplement: Attachment — Submitted filename: Response to Reviewers_R3.docx [file pone.0312205.s004.docx]

Dr. Brian M. Ward

Academic Editor

PLOS ONE

August 5, 2024

**Manuscript reference number: PONE-D-23-30862R2**

**Title: A novel oncolytic vaccinia virus with multiple gene modifications involved in viral replication and maturation increases safety for intravenous administration while maintaining proliferative potential in cancer cells**

Dear Dr. Ward:

Thank you for your email dated June 24, 2024, enclosing the reviewers’ comments. We appreciate your kind suggestion about resubmission and the constructive comments made by you and the reviewers. We have carefully reviewed the comments and have revised the manuscript accordingly. Our responses are given in a point-by-point manner below. Other minor corrections are reflected in the ‘Revised Manuscript with Track Changes’ file.

We hope the revised version is now suitable for publication and look forward to hearing from you in due course.

Go Okita

KM Biologics Co., Ltd.

**RESPONSES TO REVIEWERS’ COMMENTS**

**Journal requirements:**

*In particular:*

- *Both reviewers have pointed out the lack of statistics in critical pieces of data. This was previously requested and not addressed. Statistical analysis on the designated data will need to be done before the manuscript can be accepted.*
- *Both reviewers found the figures to be sub-standard. Please fix accordingly.*
- *One of the reviewers has pointed out that the actual viruses created using the Bac system were not genetically verified. This is a critical step to be sure the viruses used in the manuscript have the genotypes desired.*

**Our responses:**

- We added the mean value ± SEM to the duplicate data (Figs. 4 and 5), expressed the animal survival rate in a Kaplan-Meier plot, and performed a log-rank test to test for significance (Fig 6).
- To make it easier for readers to understand, we have added color coding and indications to the types of viruses in the figures. The resolution of figures such as the photo in Fig 9 has been improved. All figures were confirmed to be free of problems using the PACE digital diagnostic tool.
- We added that the base sequence of the DNA extracted from the modified virus was confirmed to be as designed for the modified region. The discussion stated that confirmation of the entire viral genome sequence is important for future drug development.

**Comments from Reviewer #4**

*- From an experimental perspective my only major concern is that the authors have not genome sequenced their viruses. This is a concern because if one uses a homologous poxvirus to reactivate another poxvirus, there is a significant risk of recombination between the transfected and infecting DNA. Simply screening for loss of the helper-virus encoded GFP plus some PCR won't necessarily confirm the identity of a virus as there may be short patches of one virus introgressed into another. Maybe the PCR data is good enough, but given how easy it is to sequence poxviruses nowadays, I'd strongly suggest that the authors use whole genome sequencing to confirm the identity of each strain.* *The method will also detect randomly scattered mutations arising during the various manipulations and which are not detected by PCR.*

**Our responses:**

We added that the base sequence of the DNA extracted from the modified virus confirmed that the modified region was as designed.

We recognize the importance of whole-genome analysis of engineered viruses, so we have added the following to the Discussion: “Furthermore, this study revealed that it is also possible to use a virus lacking F4L as a helper virus to suppress the proliferation of LC16m8. When producing modified viruses as pharmaceuticals in the future, it is important to use whole genome sequencing to confirm that there are no mutations in the gene sequence from virus seed to the end of virus culture.”

*- BTW, it would be good to cite (PMID: 11570497) as it provides an earlier example of using a vaccinia to reactivate another vaccinia albeit with a different trick to the method.*

**Our responses:**

The paper you introduced has been added as a new reference number 17.

*- I'd also note that Figure 6 is a Kaplan-Meier plot, but it's not correctly drawn that way nor properly analyzed using statistics.*

**Our responses:**

For Fig 6, we correctly created a Kaplan-Meier plot of animal survival and performed a log-rank test to test significance.

*- Only Fig 3 has any attached statistics, in fact all of graphs need further analysis.*

**Our responses:**

We added the mean value ± SEM to the duplicate data (Figs. 4 and 5).

*- Some of the speculation in the discussion concerns matters previously investigated in ref. 13, but not here properly cited. This includes the interactions with cellular RR subunits. Whether a delta F4L virus can be used as an oncolytic virus has also been tested but is again not cited.*

**Our responses:**

We have added the content of the cited paper you pointed out and inserted the past research on oncolytic viruses with F4L deleted as a new reference number 15.

*- p12 (line 87) - "highly homologous".* *Use "similar" where a degree of similarity needs to be mentioned.*

**Our responses:**

We have corrected it as you pointed out.

*- p12 (line 100) - vaccinia encodes two genes for the RR.*

**Our responses:**

We have corrected it as you pointed out.

*- p17 (line 172) - Suggest indicating that black arrows are genes from IHD-J.*

**Our responses:**

We have added it as per your suggestion.

*-* *pp17-19 - Suggest condensing and/or deleting much of the description of plasmid and BACmid construction.*

**Our responses:**

We have significantly shortened the description of the Construction of modified BACmid in Materials and Methods and added the published patent with a detailed description as a new document number 19.

*-* *p. 22 (line 269) - coli not Coli.*

**Our responses:**

This description has been removed.

*-* *p. 32 (line 426) - Some journals don't permit "data not shown".*

**Our responses:**

This description has been removed.

*-* *p. 33 (line 442-443) - It's much easier to do the math for the reader. That is 8x10^4 and 4x10^5 PFU. The MOI must also be reported based on cell counts at the time. The preferred approach would be to plot cytotoxicity over a range of MOI's, not just pick a narrow (5-fold) window where the effects are perhaps most exaggerated.*

**Our responses:**

Because the virus was inoculated after culturing under serum starvation conditions following cell adhesion, cell numbers were not counted at that point. The unit has been corrected to PFU/well to express the amount of virus inoculated into each cell.

*- p. 33 (line 447) - There's something missing where it says "(data labels)".*

**Our responses:**

This description has been removed.

*- p. 42 (line 597-598) - See comment regarding lines 442-443.*

**Our responses:**

Please see the response above.

*-* *Figures 4 and 5. The Y axis should be labelled as x10^-4 (or -6 or -7). The label indicates what math operation was applied to the actual data to create the axis numbering. In such experiments the titers (e.g. 4x10^4 PFU/mL) would have been multiplied by 10^-4 to plot them.*

**Our responses:**

The notation method of y-axis values has been corrected.

*-I'm not convinced Figure 9 adds anything to the paper as most expert readers would be familiar with mouse pathology.*

**Our responses:**

For readers not familiar with mouse pathology, we have visualized the viral symptom scores. The resolution of the photos in Fig 9 has been improved.

**Comments from Reviewer #5**

*-* There is a lack of statistical analysis, particularly as it relates to Figure 4 and 5. It looks like there may be statistical differences. Were statistics conducted? If so, statistical analysis should be included and described. If not, why were statistical analysis not completed?

**Our responses:**

In Figures 4 and 5, mean values ± SEM have been added to the duplicate data. Due to the small data size (n=2), tests for differences in means were not performed.

*-* *Figure 9: the resolution of the mouse photographs is poor. The viral symptoms that the authors note are present, need to be indicated more clearly. It is not clear what exactly we are looking at.*

**Our responses:**

The resolution of the photos in Fig 9 has been improved. We hope you can see that there is one pox in the tail in the Score 1 photo.

*-* *In general, figures could be better labeled so as it make it easier for the reader to follow what is being shown. In particular, a legend for the different colors and shapes should be provided within the figure for Figures 6, 7, and 8. While it is laid out in the figure legend, it is difficult to follow. Furthermore, labeled data bars with the virus being used rather than with letters, would make it easier to follow.*

**Our responses:**

Following your kind suggestion, we have color-coded the types of viruses in these figures to make it easier for readers to understand.

*-* *line 18 in the abstract: consider changing "high safety" to "improved safety"*

**Our responses:**

We have corrected it as you pointed out.

*-* *Reference to normal cells is present throughout the manuscript. What is the definition of a "normal" cell? This is not well-defined.*

**Our responses:**

Throughout the manuscript, we have specified that the normal cells used in this study are NHDF.

*-* *There are a lack of citations throughout the Introduction where citations should be present. For example, line 63, 66. Ensure citations are present where prior work is being cited or referenced.*

**Our responses:**

The paper related to the description from lines 63 to 66 has been inserted as a new reference number 3.

*-* *Line 345 and 350: "centrifugal supernatant" is not clear. Modify the wording. Is this supposed to read, supernatant centrifuged?*

**Our responses:**

We have corrected it as you pointed out.

*-* *Line 415: how do you define "many smallpox lesions"? Is there a number?*

**Our responses:**

We corrected "many" to "more than 4".

*-* *Line 463: Reference to "data labels" is unclear. Please be more specific.*

**Our responses:**

This description has been removed.

*-* *Line 545: This statement "as an animal symptom due to virus administration" is unclear.*

**Our responses:**

This description has been removed.
